# Merging 3D optical measurement system (structured light-based surface topography) and digital radiograms - the technique and preliminary results

**DOI:** 10.1186/1748-7161-8-S2-O24

**Published:** 2013-09-18

**Authors:** Glinkowski Wojciech, Paśko Sławomir, Walesiak Katarzyna, Sitnik Robert, Górecki Andrzej

**Affiliations:** 1 Chair and Department of Orthopaedics and Traumatology of Locomotor System, Center of Excellence “TeleOrto”, Baby Jesus Clinical Hospital, Medical University of Warsaw, Poland

## Background

Diagnosing scoliosis requires the ability to visualize curvature of the spine in a 3-dimensional (3D) environment. 2-dimensional X ray remains the primary imaging modality[1]. However, recent progress in 3D-optical imaging may improve diagnostics and increase its safety for scoliosis patients[2].

## Purpose

The purpose of this study was to present the potential usefulness of merging data from the 1-directional radiogram with a 3D model obtained with an optical measurement system.

## Methods

The images of 11 adolescents (average age 15.05 years) with adolescent idiopathic scoliosis (AIS) were selected for this study. Average Cobb angle 31.7° surface kyphosis angle 10.45° and surface lordosis angle 36.06°. An IRB was approved for this study. The merging algorithms were developed as an operational plug-in for OsiriX Imaging Software used in the facility for viewing medical images from the hospital PACS system. The 3D data from a 4-directional (360°) 3D optical measurement system (structured light-based surface topography) and digital radiograms were merged into one, consistent 3D model. The plug-in allowed loading and adjusting the data collected by these two systems. Finally, the composed 3D image was viewed, processed and saved in DICOM file format.

## Results

Merged images showed the data obtained from a 4-directional 3D optical system and X ray for the same patient made the same day. The results of this comparison are presented in graphical form. Additionally, patients appreciated that the merged 3D/X ray images provided a better understanding of the deformity and the ability to see their body surface from a new, unexpected perspective.

## Conclusions and discussion

Merging of data obtained with the 4-directional optical measurement system with data taken from another medical system, such as X ray photography, gives physicians a powerful diagnostics tool that, combines the advantages of both examination methods[3]. Moreover, as acquisition time for 4-directional optical measurement is short (a few seconds) and there is no X ray radiation, the examination can be repeated as many times as needed.
